# Analysis of heroin effects on calcium channels in rat cardiomyocytes based on transcriptomics and metabolomics

**DOI:** 10.1515/med-2023-0765

**Published:** 2023-07-31

**Authors:** Liping Su, Li Liu, Min Ji, Xiayun Hu, Min Liang, Ziyang Lu, Zhiguo Wang, Yaling Guan, Jinling Xiao, Mengjie Zhuang, Sensen Zhu, Long Yang, Hongwei Pu

**Affiliations:** Department of Pathology, First Affiliated Hospital, Xinjiang Medical University, Urumqi, Xinjiang, PR China; School of Basic Medicine, Xinjiang Medical University, Urumqi, Xinjiang, PR China; Department of Pathology, Shanghai Changhai Hospital, Shanghai, China; Discipline Inspection and Supervision Department, First Affiliated Hospital, Xinjiang Medical University, Urumqi, Xinjiang, PR China; Department of Pathology, Qilu Hospital, Jinan, Shandong Province, PR China; Xinjiang Hengzheng Judicial Expertise Center, Urumqi, China; Department of Anesthesiology, First Affiliated Hospital, Xinjiang Medical University, Urumqi, Xinjiang, PR China; Department of Discipline Construction, First Affiliated Hospital, Xinjiang Medical University, No. 137, Liyushan South Road, Urumqi 830054, Xinjiang, PR China

**Keywords:** heroin, transcriptomics, action potential, L-calcium channel, arrhythmia

## Abstract

Heroin can cause damage to many human organs, possibly leading to different types of arrhythmias and abnormal electrophysiological function of the heart muscle and the steady state of calcium-ion channels. We explored cardiomyocytes treated with heroin and the effect on calcium-ion channels. Transcriptomics and metabolomics were used to screen for differential genes and metabolite alterations after heroin administration to jointly analyze the effect of heroin on calcium channels in cardiomyocytes. Cardiomyocytes from primary neonatal rats were cultured *in vitro* and were treated with different concentrations of heroin to observe the changes in morphology and spontaneous beat frequency and rhythm by a patch clamp technique. Transcriptomic studies selected a total of 1,432 differentially expressed genes, 941 upregulated and 491 downregulated genes in rat cardiomyocytes from the control and drug intervention groups. Gene Ontology functional enrichment showed that 1,432 differential genes selected by the two groups were mainly involved in the regulation of the multicellular organismal process, response to external stimulus, myofibril, inflammatory response, muscle system process, cardiac muscle contraction, etc. Kyoto Encyclopedia of Genes and Genomes pathway enrichment analysis indicated that these genes were mainly concentrated in cardiac muscle contraction, osteoclast differentiation, adrenergic signaling in cardiomyocytes, dilated cardiomyopathy, hypertrophic cardiomyopathy, and other important pathways. Metabolomic testing further suggested that cardiomyocyte metabolism was severely affected after heroin intervention. After the treatment with heroin, the L-type calcium channel current *I*–*V* curve was up-shifted, the peak value was significantly lower than that of the control group, action potential duration 90 was significantly increased in the action potential, resting potential negative value was lowered, and action potential amplitude was significantly decreased in cardiomyocytes. In this study, heroin could cause morphological changes in primary cardiomyocytes of neonatal rats and electrophysiological function. Heroin can cause myocardial contraction and calcium channel abnormalities, damage the myocardium, and change the action potential and L-type calcium channel.

## 1 Introduction

In recent years, the abuse and addiction to heroin have become a serious public health concern in society. Heroin has a strong dependence and can cause various degrees of damage to many human organs [1]. A large number of studies have shown that heroin addiction has serious effects on the cardiovascular system, can induce various arrhythmias, and cause intracellular calcium overload, affecting the normal physiological function of the heart [2,3]. Cardiac electrophysiological activity is closely related to myocardial calcium channels, and calcium channels are regulated by various calcium-dependent regulatory proteins. Calcium ion (Ca^2+^) is an important second messenger in cells, which mainly maintains the normal electrophysiological function of the cell membrane and ensures the normal progress of various biological signal transduction pathways. Intracellular Ca^2+^ overload in cardiomyocytes is a major feature of cardiomyocyte injury. The prolongation of the action potential duration (APD) of ventricular myocytes can enhance intracellular calcium currents and change myocardial contractility. At the same time, changes in calcium related proteins can also cause abnormal changes in action potential, leading to the occurrence of cardiac arrhythmias. It plays an important role in the signal pathway mediated by opioid receptors [4,5]. Exploring the relationship between calcium ion and diacetylmorphine arrhythmia can provide new thinking for finding new drug targets and scientific prevention and treatment in clinical patients with diacetylmorphine arrhythmia.

## 2 Materials and methods

### 2.1 Materials

The 3-day-old SPF-class Sprague-Dawley (SD) neonatal rats were provided by the Experimental Animal Center of Xinjiang Medical University (Experimental animal ethical approval of the Medical Ethics Committee of the First Affiliated Hospital of Xinjiang Medical University: K201907-06). Heroin was provided by the Anti-Drug Corps of Xinjiang Uygur Autonomous Region. Anti-cardiac troponin T(ab209813) was provided by Abcam company, USA, secondary antibody to mouse (goat polyclonal secondary antibody to mouse IgG H&L) was provided by Alexa Flour^®^ 488, and ab150113 was provided by Abcam company, USA.

### 2.2 Cell culture

One hundred and twenty SD neonatal rats (1–3 days) were euthanatized, and their skin was disinfected with 75% alcohol under aseptic conditions. After cutting approximately half of the heart, the remaining heart was cut using an ophthalmic scissor into 7–8 petals along the tip of the heart in the radial shape. Then, 0.025% trypsin was added for digestion, and 0.6 mg/mL of type II collagenase was added for vibrating digestion for 7 min. Then, centrifugation was performed at 1,000 rpm for 5 min. The cells were counted and inoculated in a 100 mm culture dish at a cell density of 5 × 10^5^ cells/mL. In this way, the proliferation of cardiac fibroblasts was inhibited. The cells were linked and beaten at the same frequency after being cultured for 5–6 days. Then, they could be used for subsequent experiments.

### 2.3 Immunofluorescence

The cultured primary cardiomyocytes were seeded or placed in a polylysine-pretreated laser confocal culture dish. The cells were fixed with 4% tissue cell fixative for 40 min at room temperature. Then, 0.3% Triton X-100 working solution was added to the culture dish for perforation, followed by incubation for 30 min. The anti-cardiac troponin T (1:500) was diluted with 0.3% Triton X-100 working solution. Bovine serum albumin was used for blockage for 1 h and incubated with the primary antibody overnight at 4℃. Then, it was rinsed with phosphate-buffered saline (PBS) three times for 5 min. The secondary antibody, which is the FITC-labeled goat anti-mouse IgG antibody (goat antibody to mouse IgG H&L [Alexa Flour^®^ 488], 1:500), was incubated for 1 h at 37℃ in the dark. DAPI was used to stain the nucleus for 10 min. Under the confocal microscope, the purity of cardiomyocytes was identified and observed by taking five different visual fields. The expression of α-actin was observed at the excitation wavelength of 488 nm, and DAPI staining was observed under the excitation of ultraviolet wavelength. The positive cells were expressed, and the proportion of positive cells was counted by the two lasers that were superimposed at the same time.

### 2.4 Cell morphology

A total of 10^−4^ mol/L heroin was used for the intervention with cardiomyocytes. The concentration of heroin was 0 mol/L (N group) and 10^−4^ mol/L (H group). After the PBS solution in equivalent volume was added to the normal group and acted for 24 h, the changes in morphology and spontaneous beat frequency and rhythm of cardiomyocytes were observed under an inverted fluorescence microscope at different concentrations of drug interference.

### 2.5 Sample intervention and collection

Cultured primary rat cardiomyocytes were divided into the normal control group (Con) and drug intervention group (Drug). The Drug group was given 10^−4^ mol/L heroin for 24 h according to the optimal drug action concentration determined by a previous experiment, and an equal amount of PBS was added to the Con group. After 24 h, the cells were collected in 1.5 mL Eppendorf tubes, repeating each group for subsequent transcriptomics and targeted metabolomics experiments based on the multiple reaction monitoring (MRM) method.

### 2.6 Transcriptomic detection and analysis

Transcriptomic detection and analysis involved the following steps.

(1) RNA extraction: the basic principle of extraction is to prevent RNA degradation in the process of extraction, maximize the efficiency of RNA extraction, and ensure the extraction of high-quality RNA with good integrity and high purity from the target sample. (2) Sample testing: high-quality RNA is the basis for the success of the whole project. We inspected the quality of the samples to ensure the accuracy of sequencing data, and the test results could only meet the requirements of sequencing and database construction. Sample test results are shown in the quality inspection report. (3) Library construction: after the sample test was qualified, magnetic beads with oligo (dT) were used to enrich the mRNA of eukaryotes. The fragmentation buffer was subsequently added to randomly interrupt the mRNA. Using mRNA as a template with six-base random primers (random hexamers), the second strand of cDNA was synthesized by buffer and dNTPs and DNA polymerase I, and the double-stranded cDNA was subsequently purified using AMPure XP beads. Purified double-stranded cDNA was then subjected to end repair, a tail addition, and sequencing joint ligation, followed by fragment size selection with AMPure XP beads. Finally, polymerase chain reaction enrichment was performed to obtain the final cDNA library. (4) Library quality control: after the library construction was completed, the insert length (insert size) and the effective concentration of the library were tested to ensure the library quality. (5) Computer sequencing: after the library inspection was qualified, different libraries were pooled according to the target data amount and computer sequencing. (6) Bioinformatics analysis included GO analysis, KEGG analysis, protein interaction network analysis, and transcription factor analysis.

### 2.7 Analysis of targeted metabolomics tests based on MRM

Energy metabolites were analyzed in rat cardiomyocytes based on the MRM method, and all samples were prepared as QC samples using QC samples to evaluate the stability and reproducibility of the data. The relative standard deviation (RSD) results of QC samples are shown in Figure 7, where the RSD is less than 30% energy metabolism, and the data are stable and reliable in the samples. The main steps are as follows: (1) Metabolite extraction: samples were removed at −80℃, and 1 mL of methanol acetonitrile solution (2:2:1, v/v) was added. The samples were vortexed for 60 s, underwent low-temperature sonication for 30 min twice, put at −20℃ for 1 h for protein precipitation, and centrifuged at 14,000 RCF at 4℃ for 20 min. The supernatant was frozen to get dry samples at −80℃. (2) Chromatography-mass spectrometry analysis: samples were separated using an Agilent 1290 Infinity LC ultra-HPLC system. Mass spectrometry was performed using a 5500 QTRAP mass spectrometer (AB SCIEX) in negative ion mode. (3) Data processing: the spectral peak area and retention time were extracted using MultiQuant software.

### 2.8 Cell patch clamp

After the treatment with 10^−4^ mol/L heroin for 24 and 48 h, the action potential and calcium channel current were detected. After the whole-cell recording mode was formed, the voltage clamp mode was changed to the current clamp mode, and the action potential was recorded. The patch clamp system was controlled by patch clamp amplifiers EPC10 and pCLAMP8.0 (Axon Instrument) software. The digital-to-analog converter performed *I*_Ca-L_ to stimulate signal generation, feedback signal acquisition, and data analysis. The following solution formulation was used for detecting action potential: extracellular fluid: 140 mmol/L NaCl, 3.5 mmol/L KCl, 10 mmol/L HEPES, 10 mmol/L glucose, 1.25 mmol/L NaH_2_PO_4_, 1 mmol/L MgCl_2_, 2 mmol/L CaCl_2_, NaOH, and pH = 7.4. Intracellular fluid: 5 mmol/L NaCl, 140 mmol/L KCl, 0.1 mmol/L CaCl_2_, 1 mmol/L MgCl_2_, 10 mmol/L HEPES, 2 mmol/L Mg-ATP, KOH, and pH = 7.2. The following solution formulation was used for detecting calcium channels: extracellular fluid: 140 mmol/L TEA-Cl, 2 mmol/L MgCl_2_, 10 mmol/L HEPES, 10 mmol/L glucose, CsOH, and pH = 7.4. Intracellular fluid: 120 mmol/L CsCl, 1 mmol/L MgCl_2_, 10 mmol/L HEPES, 4 mmol/L Mg-ATP, CsOH, and pH = 7.2.

### 2.9 Statistical analysis

The statistical results concerning measurement data and enumeration data were tested by SPSS 23.0 software. Data were presented as the mean value ± standard error of the mean from at least three independent experiments performed in duplicate or triplicate. Statistical analysis was performed by one-way ANOVA with Bonferroni post hoc test, and the test level was set at *α* = 0.05.

## 3 Results

### 3.1 Purity identification for primarily cultured cardiomyocytes of neonatal rats

**Figure 1:**
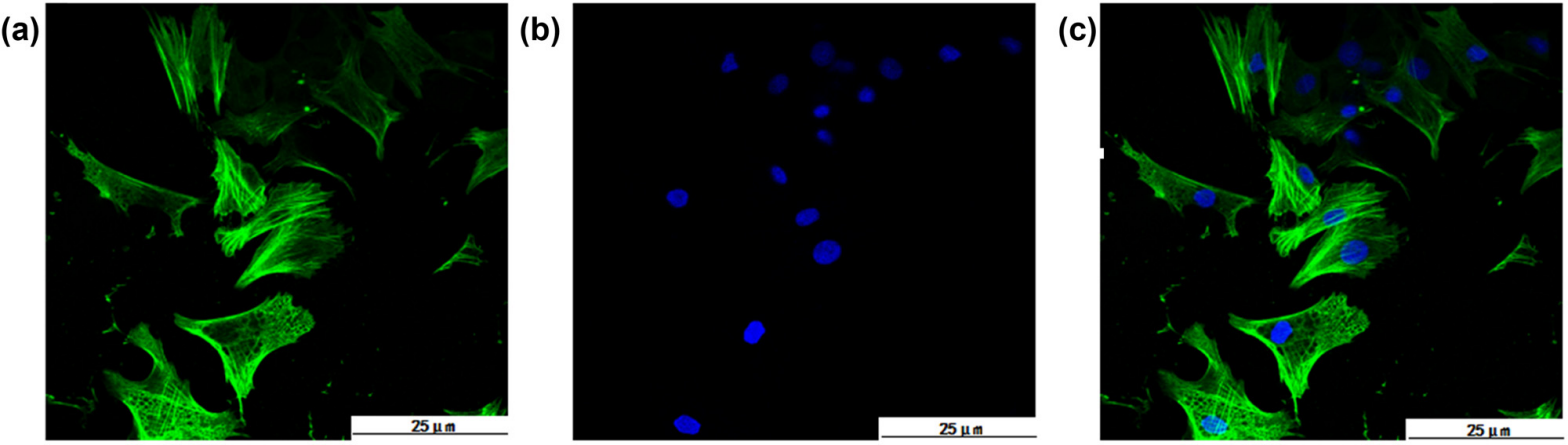
Identification results of primary cardiomyocytes. (a) Anti-cardiac troponin T cytoplasm stained green; (b) DAPI nuclei are blue; and (c) A + B synthesis.

The passaged-7 primarily cultured cardiomyocytes were identified by laser confocal microscopy. The expression of α-actin, which is a classical biomarker specific to cardiomyocytes, was detected in the primary cardiomyocytes. After the primary antibody FITC-labeled goat anti-mouse IgG combined with the myocardial cell-specific protein cardiac troponin T in the cytoplasm, the grid-like or filamentous-green fluorescence (Figure 1b) was excited by a 488 nm laser. DAPI dying was used to locate the nucleus, and it formed circular or elliptical blue fluorescence (Figure 1a) excited by the ultraviolet laser. The overlap figures showed the intact morphological structure of primary cardiomyocytes with various forms, including triangles, fusiforms, and polygons. The actin protein was filamentously distributed and interwoven into a network in the cells (Figure 1c). Five randfluorescent fields were selected from each dish, and cells expressing blue and green fluorescence were identified as positive cells. The number of blue fluorescence was recorded as the total number of cells, and the proportion of positive cells was calculated. The results showed that the purity of the primary cultured rat cardiomyocytes was >90%, which could be used in subsequent experiments.

### 3.2 Morphological changes in cardiomyocytes treated with different concentrations of heroin

**Figure 2:**
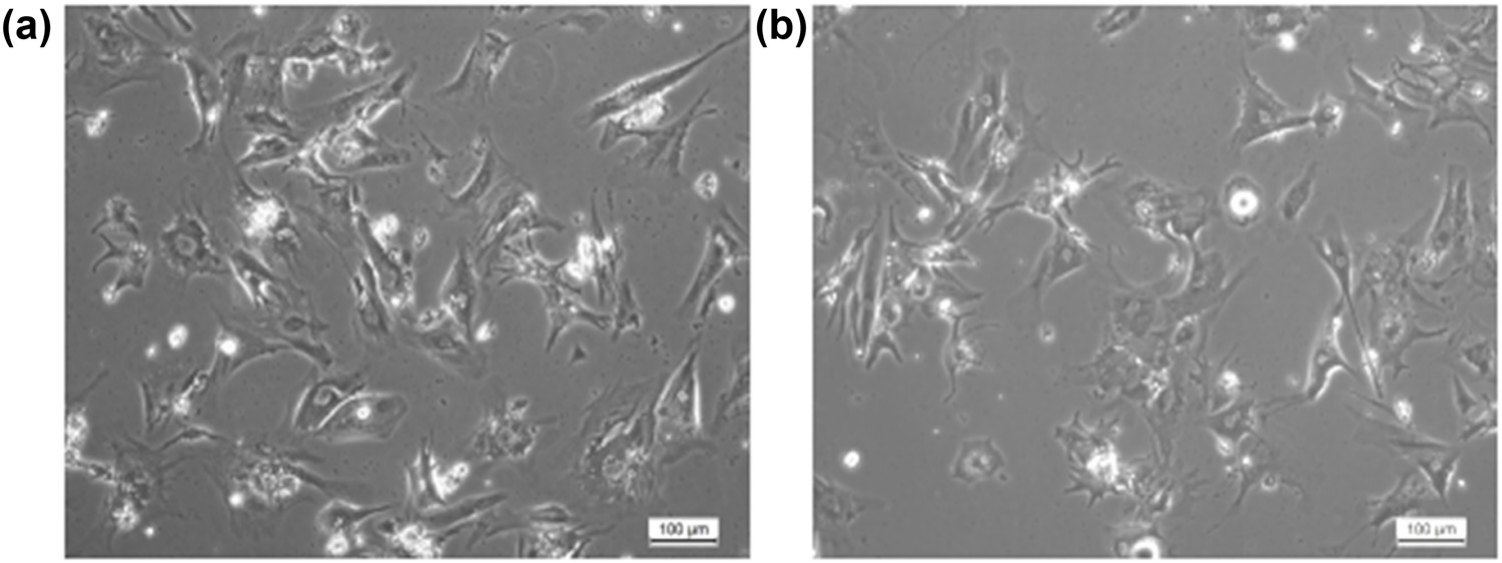
Morphological changes in myocardial cells after heroin intervention for 24 h. (a) Normal primary cardiomyocyte group; (b) Diacetylmorphine intervention group.

When the cardiomyocytes of primary neonatal rats were continuously cultured for 3 days, spontaneous pulsation occurred, and the pulsation frequency was slower. Then, the cells were cultured for 7 days, and most cells recovered to spontaneous pulsation, while some of the cardiomyocytes were connected into pieces and beaten with a regular rhythm. In the control group with PBS, the quantities of cultured cardiomyocytes were large, and their morphology was intact. Additionally, the cell morphology was diverse and triangular, long fusiform, and polygonal, with a clear structure, intact membrane, and bright membrane edge, and the nucleus was round or similar to round. The size was normal, the nuclear membrane was smooth, and the cells were connected to each other by pseudopods (Figure 2a). With the intervention of heroin, the number of cardiomyocytes sharply decreased, the cell membrane shrank, the pseudopods decreased, the nuclear structure was blurred, and the cell debris appeared in the cells (Figure 2b).

### 3.3 Transcriptomic screening for differential genes and signaling pathway changes before and after heroin intervention

**Figure 3:**
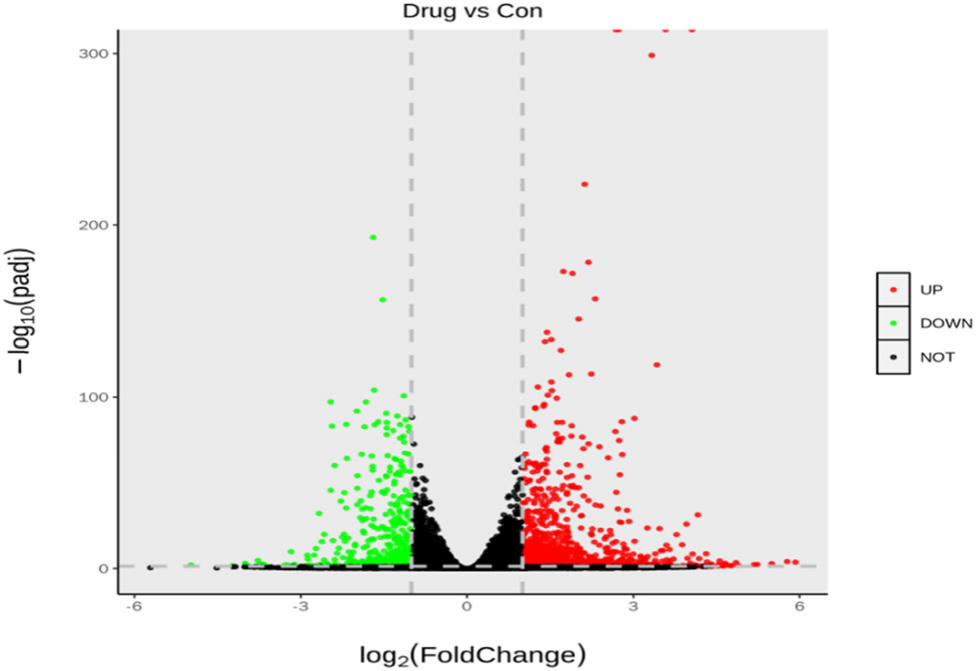
Volcano plot of the differential gene expression distribution. The abscissa indicates the fold change of gene expression in different experimental groups or in different samples; the ordinate indicates the statistically significant degree of gene expression change. Scatters in the plot represent individual genes, black dots indicate genes with no significant differences, red dots indicate upregulated genes with significant differences, and green dots indicate downregulated genes with significant differences.

**Figure 4:**
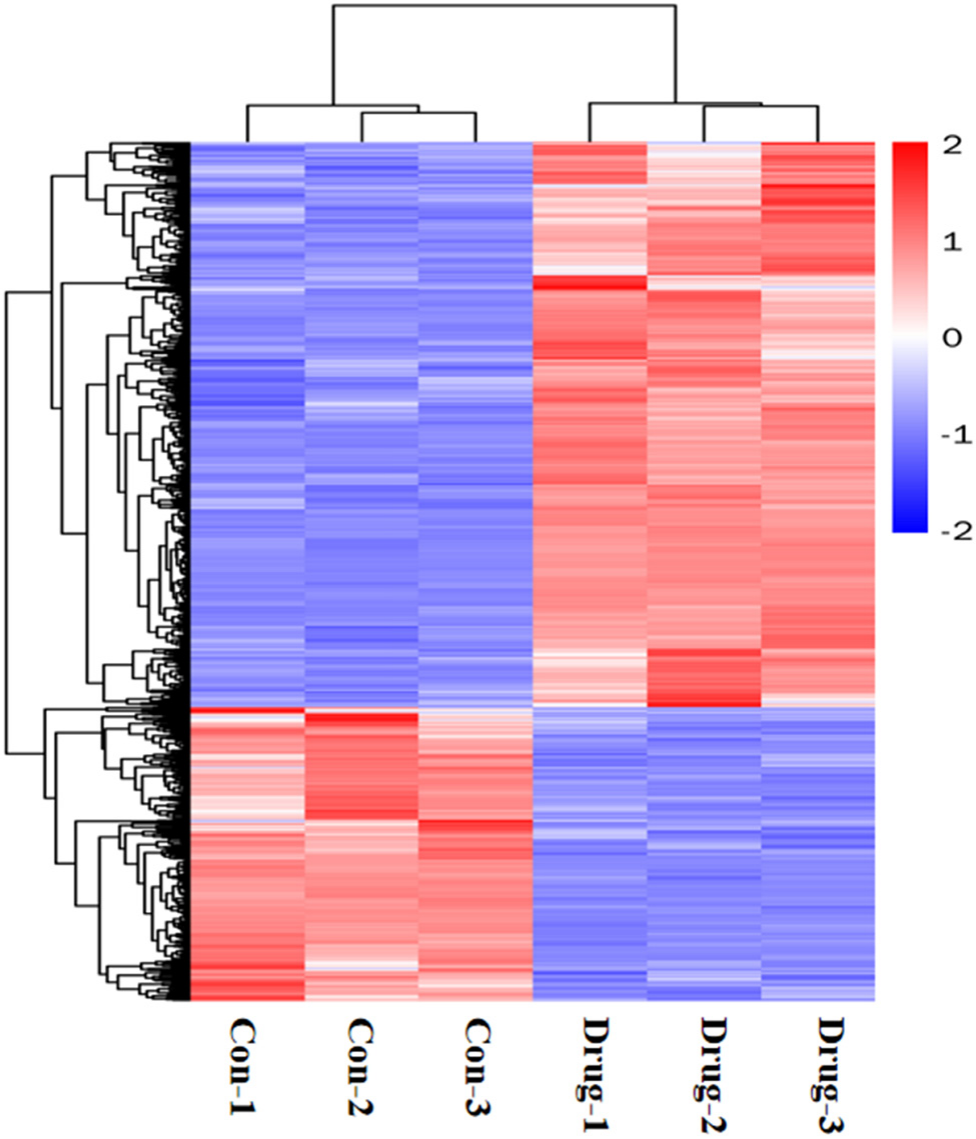
Differential gene cluster map. Each column represents one sample, and each row represents one gene. Red indicates upregulation and blue downregulation. Above is the dendrogram of the sample clusters, two The closer the sample branches are, the closer the expression pattern of all different genes between these two samples is. The dendrogram of the gene clusters is shown on the left, two The closer the gene branches are, the closer their expression level is.

**Figure 5:**
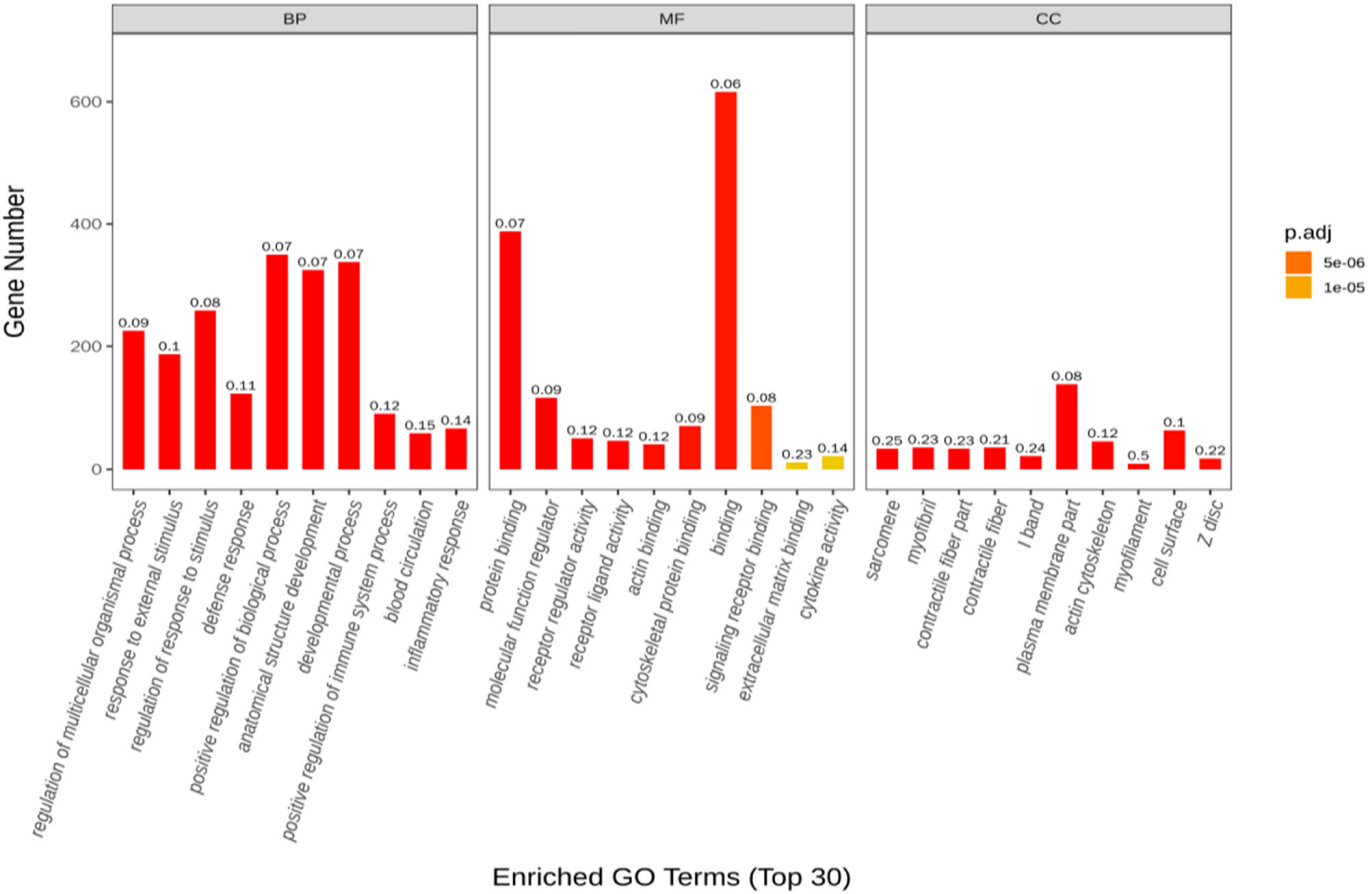
Bar graph of GO enrichment for differential genes.

**Figure 6:**
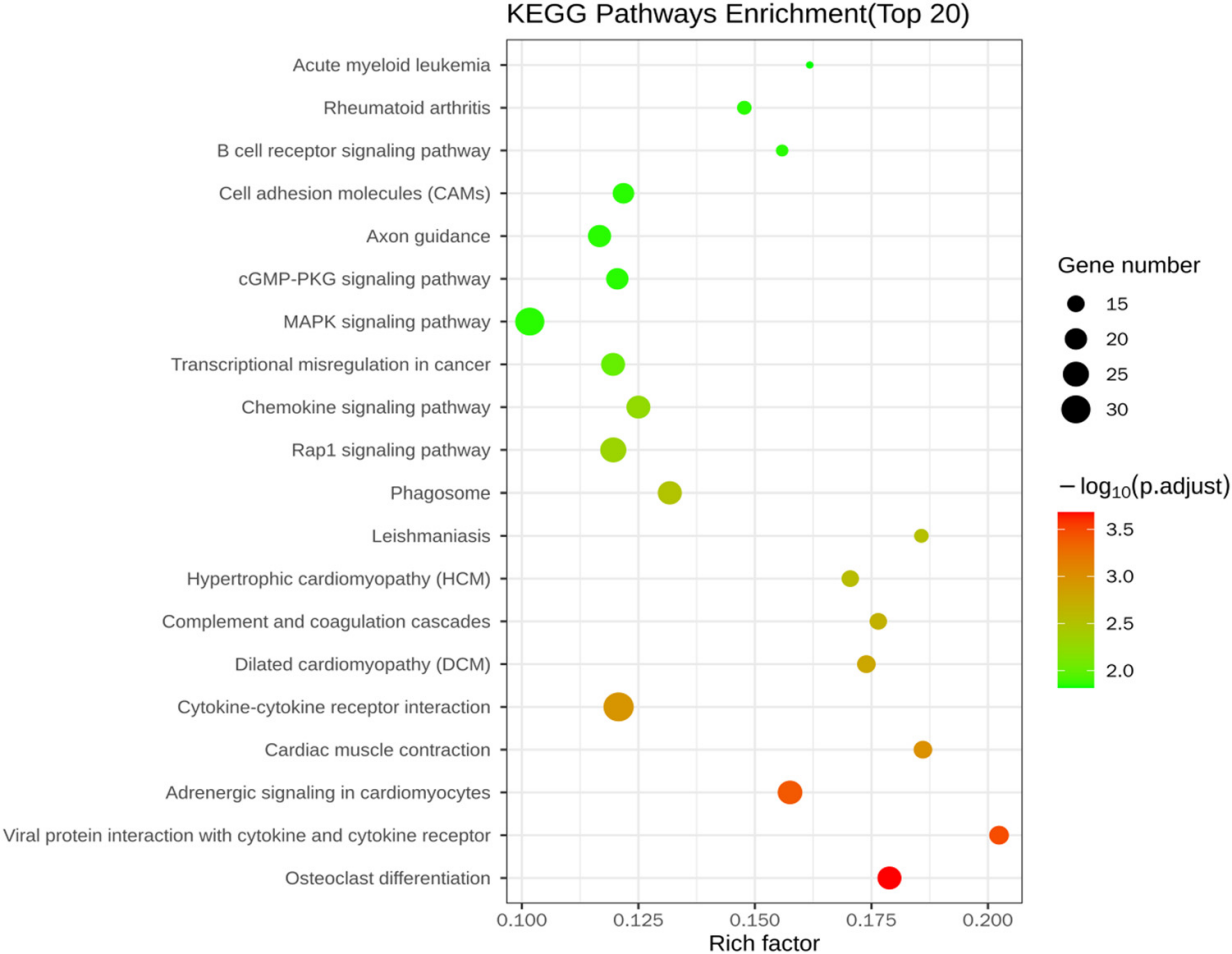
KEGG enrichment bubble plot of the differentially expressed genes.

A total of 1,432 differentially expressed genes were selected in the normal control group, 941 were upregulated and 491 were downregulated in the normal control and drug intervention groups, respectively. The direct embodiment of the gene expression level was the abundance of its transcripts, and the higher the transcript abundance, the higher the gene expression level. FPKM values for each gene in each sample were calculated using feature Counts software. FPKM represented the number of alignments to transcript per kilobase per million alignment fragments, eliminating the effect of gene length and sequencing quantity differences on the calculated gene expression. The calculated gene expression levels could be directly used to compare the gene expression differences between different samples. Table 1 shows the statistics of the number of different genes at different expression levels. A volcano plot can visually show the distribution of differential genes and their expression levels in the two groups (Figure 3). Differential gene clustering could be used to judge the changes in different genes between different groups. According to the similarity of the expression of genes in each sample, the genes were clustered to visually show the expression of genes in different samples to obtain information related to biological problems. As could be seen from the cluster map, most genes were significantly up or downregulated before and after the heroin intervention (Figure 4). We used *p* adj. of <0.05 and log 2 foldchange of >1 as the differential significance criterion. The top 30 significantly differentially expressed genes between the two groups were selected, and the analysis results are shown in Table 2. Gene Ontology (GO) functional enrichment showed that 1,432 differential genes selected by the two groups were mainly involved in the regulation of the multicellular organismal process, response to external stimulus, defense response, developmental process, myofibril, inflammatory response, circulatory system process, muscle system process, cardiac muscle contraction, cardiac muscle tissue development, etc. (Figure 5). Kyoto Encyclopedia of Genes and Genomes (KEGG) pathway enrichment analysis of 1,432 differential genes screened out the top 20 most significant differential signaling pathways. Cardiac muscle contraction, osteoclast differentiation, adrenergic signaling in cardiomyocytes, dilated cardiomyopathy (DCM), hypertrophic cardiomyopathy (HCM), and other important pathways were significantly upregulated. The NOD-like receptor signaling pathway, influenza A, TNF signaling pathway, Kaposi sarcoma-associated herpesvirus infection, herpes simplex virus 1 infection, and other important pathways were significantly downregulated (Figure 6; Table 3).

**Table 1:** Statistics of number of genes with different expression levels

Sample	0 ≤ FPKM ＜ 1	1 ≤ FPKM ＜ 5	5 ≤ FPKM ＜ 10	10 ≤ FPKM ＜ 30	30 ≤ FPKM ＜ 50	50 ≤ FPKM
Con-1	20,798 (59.56%)	4,699 (13.46%)	2,632 (7.54%)	4,018 (11.51%)	1,150 (3.29%)	1,621 (4.64%)
Con-2	20,831 (59.66%)	4,817 (13.8%)	2,586 (7.41%)	3,917 (11.22%)	1,111 (3.18%)	1,656 (4.74%)
Con-3	20,822 (59.63%)	4,769 (13.66%)	2,565 (7.35%)	3,982 (11.4%)	1,136 (3.25%)	1,644 (4.71%)
Drug-1	20,393 (58.4%)	5,135 (14.71%)	2,447 (7.01%)	4,071 (11.66%)	1,177 (3.37%)	1,695 (4.85%)
Drug-2	20,443 (58.55%)	5,148 (14.74%)	2,454 (7.03%)	4,017 (11.5%)	1,150 (3.29%)	1,706 (4.89%)
Drug-3	20,407 (58.44%)	5,073 (14.53%)	2,480 (7.1%)	4,106 (11.76%)	1,175 (3.37%)	1,677 (4.8%)

**Table 2:** Differential genes (Con vs Drug)

Number	Expression changes	Name	*p* value	Expression changes	Name	*p* value
1	Up	Reg3b	0.00000	Down	Symbol	0.00000
2	Up	Nppb	0.00000	Down	Ptx3	0.00000
3	Up	Nppa	0.00000	Down	Prg4	0.00000
4	Up	Hmox1	0.00000	Down	Rasl12	0.00000
5	Up	Lyz2	0.00000	Down	Fzd1	0.00000
6	Up	Rcan1	0.00000	Down	Apol9a	0.00000
7	Up	Gdf15	0.00000	Down	Htr2a	0.00000
8	Up	Myh7	0.00000	Down	Mx2	0.00000
9	Up	Zfand2a	0.00000	Down	Ackr3	0.00000
10	Up	Tcp11l2	0.00000	Down	Jak2	0.00000
11	Up	Xirp1	0.00000	Down	Lox	0.00000
12	Up	Gclc	0.00000	Down	Irf7	0.00000
13	Up	Myl3	0.00000	Down	F3	0.00000
14	Up	Mybpc3	0.00000	Down	AABR07059663.1	0.00000
15	Up	Myl2	0.00000	Down	Tgm2	0.00000
16	Up	Cybb	0.00000	Down	Parp9	0.00000
17	Up	Slc7a11	0.00000	Down	Samd9	0.00000
18	Up	Mtss1	0.00000	Down	Cxcl6	0.00000
19	Up	Tnnc1	0.00000	Down	3-Mar	0.00000
20	Up	Atp1b1	0.00000	Down	Prickle1	0.00000
21	Up	Casq2	0.00000	Down	Lgals3bp	0.00000
22	Up	Fhl1	0.00000	Down	Il6	0.00000
23	Up	Popdc2	0.00000	Down	Lrrc32	0.00000
24	Up	Parm1	0.00000	Down	Ahr	0.00000
25	Up	Pcf11	0.00000	Down	Grem1	0.00000
26	Up	Sorbs2	0.00000	Down	Cd55	0.00000
27	Up	Ankrd1	0.00000	Down	Ugdh	0.00000
28	Up	Osgin1	0.00000	Down	AABR07058464.1	0.00000
29	Up	Actn2	0.00000	Down	Pde7a	0.00000
30	Up	Art3	0.00000	Down	Adamts15	0.00000

**Table 3:** Differential signaling pathways (Con vs Drug)

Number	Changes	ID	KEGG pathway	Test	Ref	*p* value	FDR
1	Up	rno04260	Cardiac muscle contraction	16	86	0.00000	0.00001
2	Up	rno04380	Osteoclast differentiation	19	123	0.00000	0.00001
3	Up	rno04261	Adrenergic signaling in cardiomyocytes	19	146	0.00000	0.00014
4	Up	rno05414	DCM	14	92	0.00001	0.00040
5	Up	rno05140	Leishmaniasis	12	70	0.00001	0.00042
6	Up	rno04610	Complement and coagulation cascades	13	85	0.00001	0.00054
7	Up	rno05410	HCM	13	88	0.00002	0.00068
8	Up	rno04662	B cell receptor signaling pathway	12	77	0.00002	0.00071
9	Up	rno05150	Staphylococcus aureus infection	13	91	0.00003	0.00076
10	Up	rno04145	Phagosome	18	167	0.00004	0.00108
11	Up	rno04061	Viral protein interaction with cytokine and cytokine receptor	12	84	0.00006	0.00127
12	Up	rno04666	Fc gamma R-mediated phagocytosis	12	91	0.00012	0.00257
13	Up	rno05152	Tuberculosis	17	168	0.00015	0.00298
14	Up	rno05221	Acute myeloid leukemia	10	68	0.00018	0.00325
15	Up	rno04010	MAPK signaling pathway	24	295	0.00023	0.00391
16	Up	rno05202	Transcriptional misregulation in cancer	17	184	0.00045	0.00716
17	Up	rno04960	Aldosterone-regulated sodium reabsorption	7	39	0.00049	0.00731
18	Up	rno04514	Cell adhesion molecules	15	156	0.00064	0.00900
19	Up	rno04670	Leukocyte transendothelial migration	12	111	0.00079	0.01048
20	Up	rno05412	Arrhythmogenic right ventricular cardiomyopathy	9	74	0.00155	0.01947
1	Down	rno04621	NOD-like receptor signaling pathway	14	168	0.00000	0.00037
2	Down	rno05164	Influenza A	13	156	0.00000	0.00045
3	Down	rno04668	TNF signaling pathway	10	111	0.00003	0.00204
4	Down	rno05167	Kaposi sarcoma-associated herpesvirus infection	12	192	0.00017	0.00862
5	Down	rno05168	Herpes simplex virus 1 infection	16	321	0.00020	0.00862
6	Down	rno05160	Hepatitis C	10	150	0.00035	0.01139
7	Down	rno05165	Human papillomavirus infection	16	339	0.00037	0.01139
8	Down	rno05161	Hepatitis B	10	154	0.00044	0.01176
9	Down	rno05162	Measles	9	137	0.00078	0.01863
10	Down	rno04622	RIG-I-like receptor signaling pathway	6	64	0.00097	0.02082
11	Down	rno04620	Toll-like receptor signaling pathway	7	90	0.00113	0.02210
12	Down	rno04350	TGF-beta signaling pathway	7	95	0.00155	0.02567
13	Down	rno05169	Epstein-Barr virus infection	11	212	0.00149	0.02567
14	Down	rno04623	Cytosolic DNA-sensing pathway	5	53	0.00251	0.03859
15	Down	rno04060	Cytokine-cytokine receptor interaction	12	265	0.00288	0.04126
16	Down	rno04512	ECM-receptor interaction	6	87	0.00466	0.06256
17	Down	rno04072	Phospholipase D signaling pathway	8	151	0.00576	0.07284
18	Down	rno04657	IL-17 signaling pathway	6	94	0.00678	0.08096
19	Down	rno05146	Amoebiasis	6	99	0.00867	0.09815
20	Down	rno04933	AGE-RAGE signaling pathway in diabetic complications	6	101	0.00953	0.10243

### 3.4 Metabolomic changes in cardiomyocytes after heroin intervention

**Figure 7:**
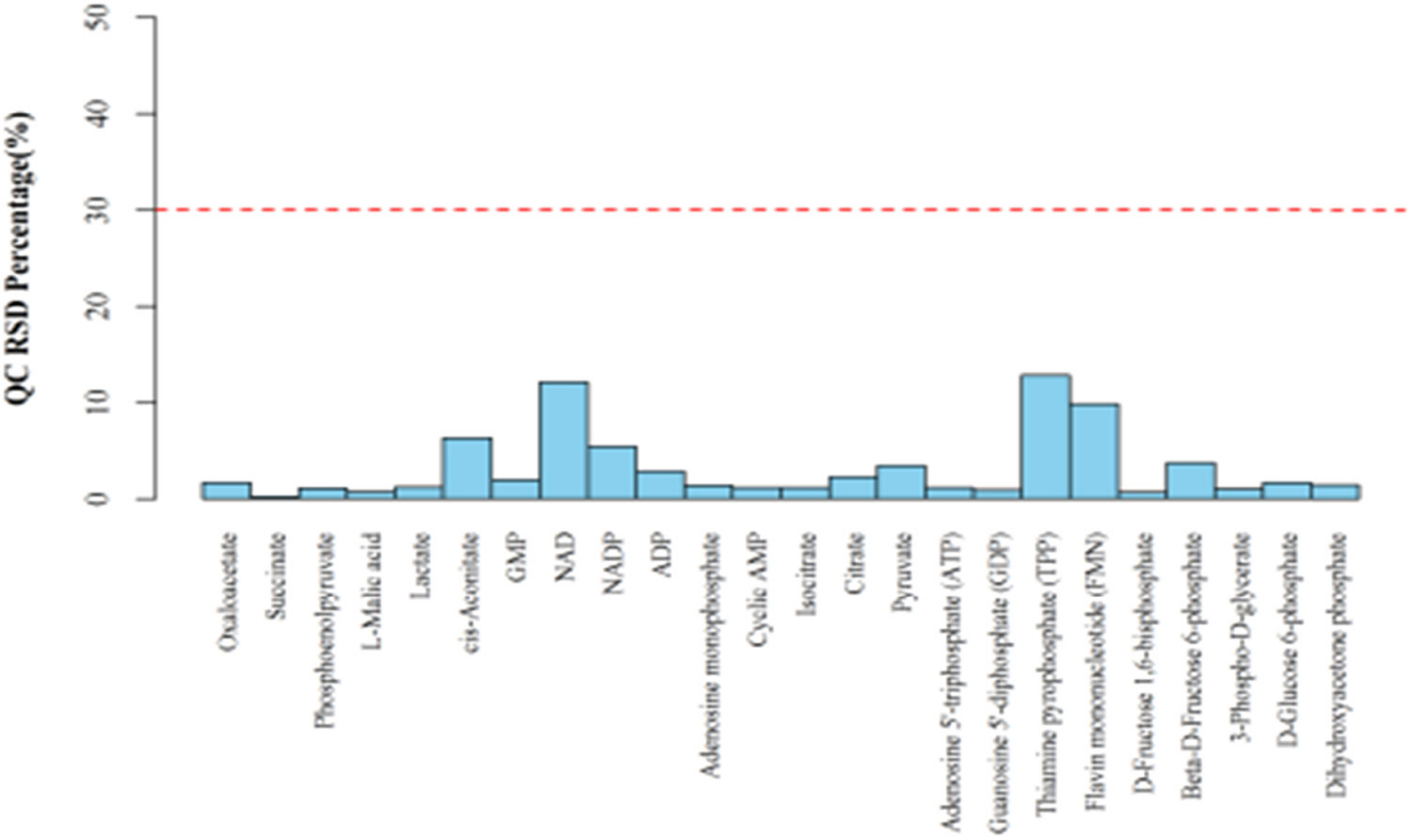
The RSD distribution of the QC samples.

**Figure 8:**
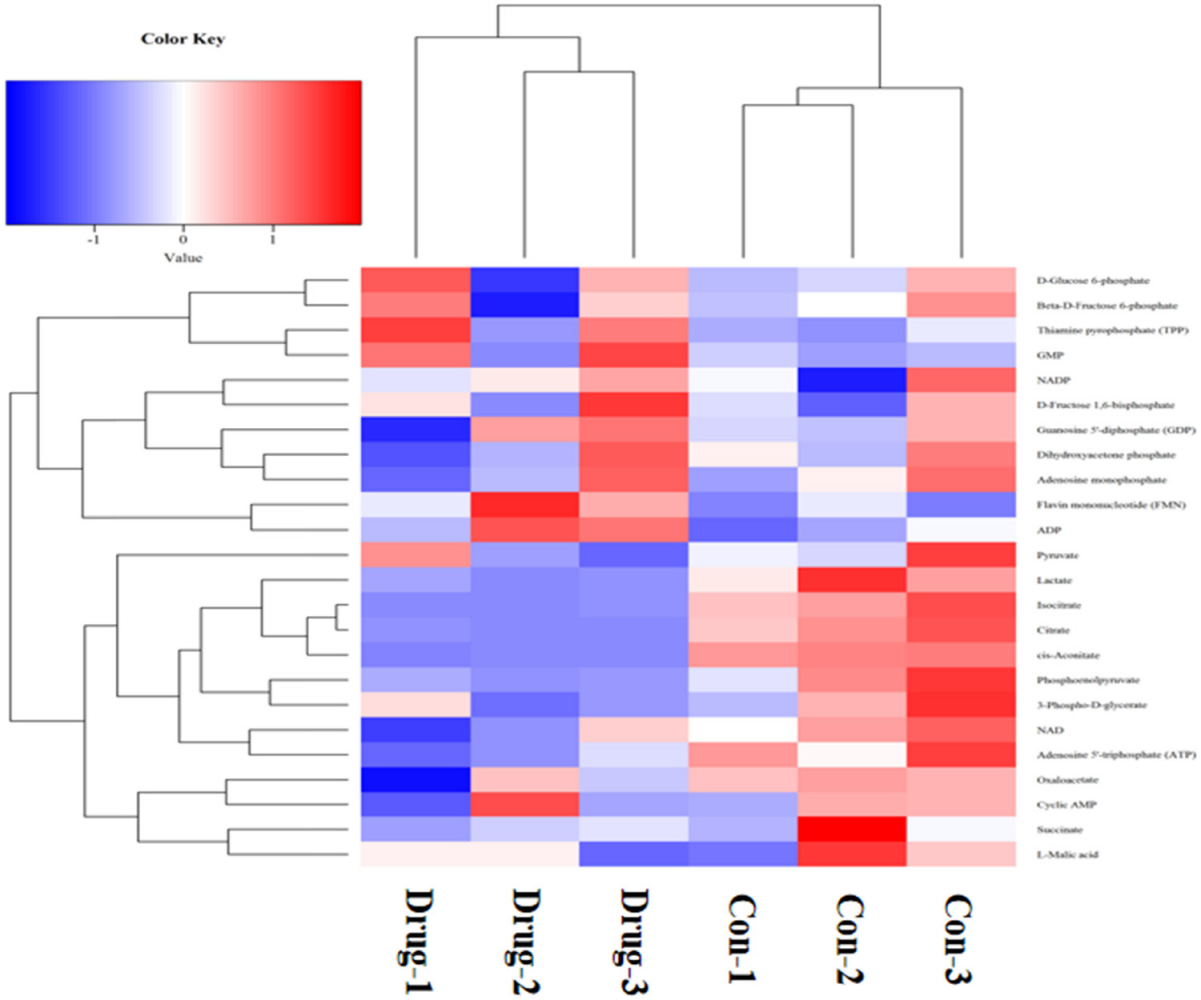
Results of the hierarchical clustering of the metabolites.

**Figure 9:**
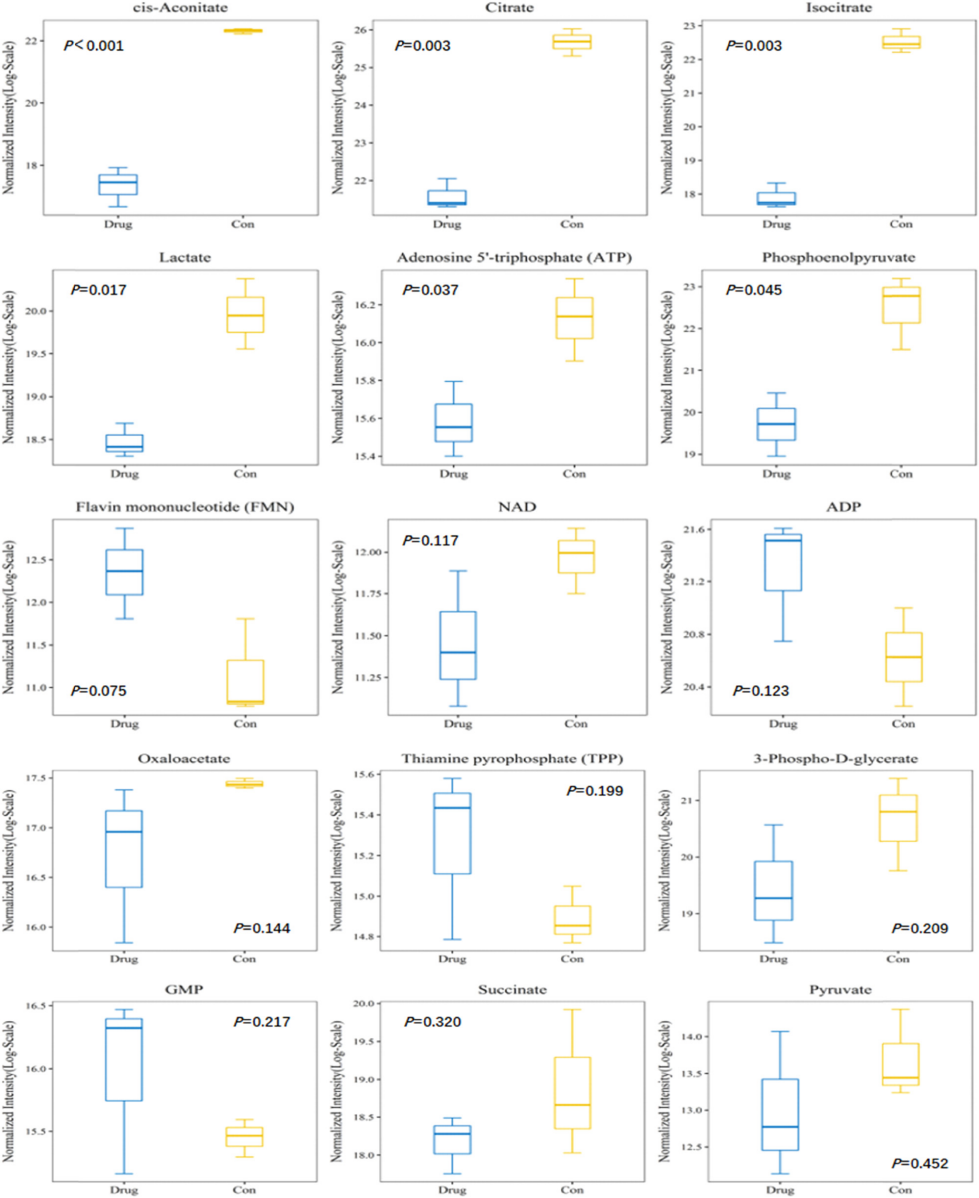

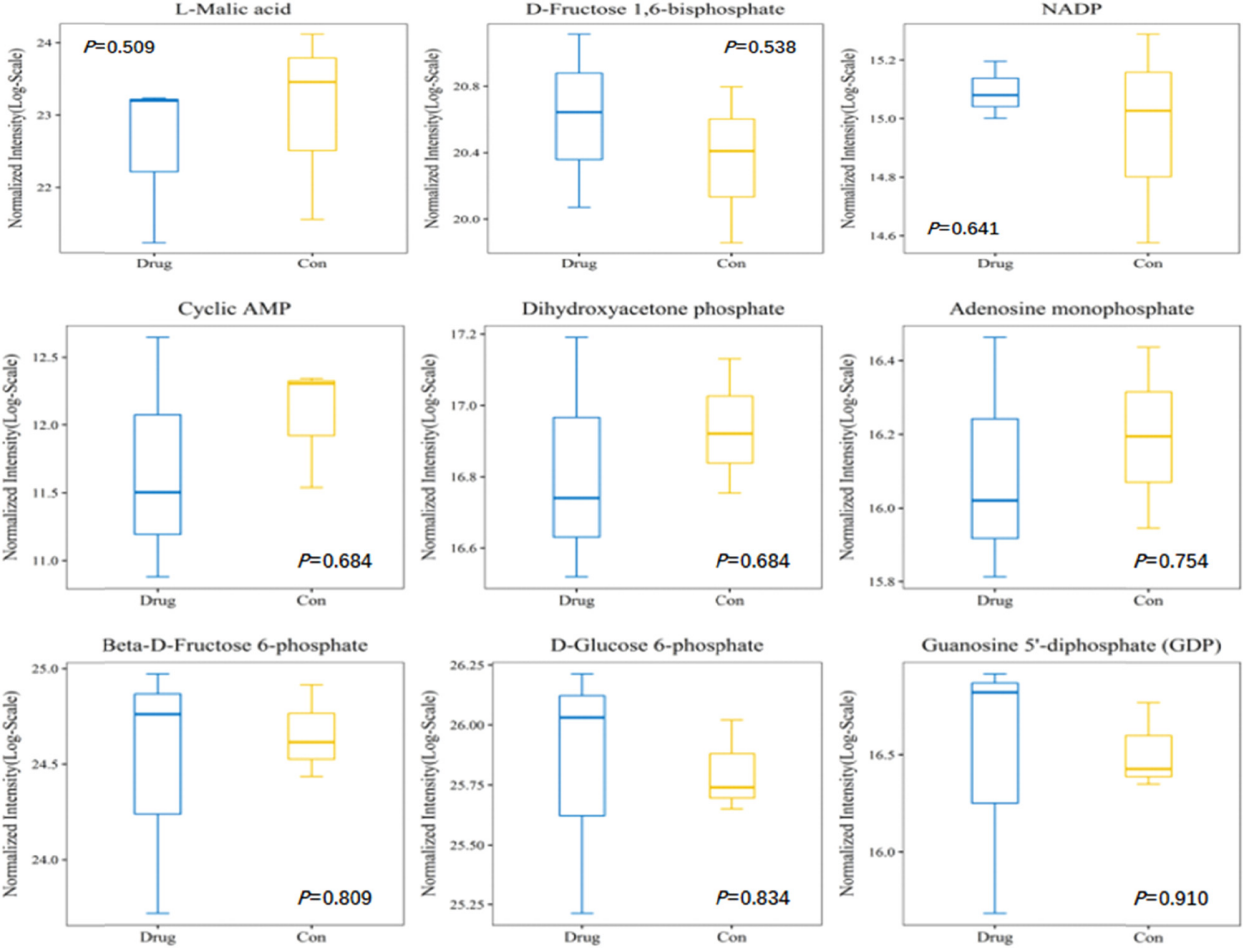
Trend diagram of group metabolite expression levels.

The results of targeted metabolomics analysis based on the MRM methods revealed significant changes in multiple metabolites in SD rat cardiomyocytes following the heroin intervention, such as cis-aconitate, citrate, isocitrate, lactate, adenosine 5′-triphosphate (ATP), phosphoenolpyruvate (all the *p*-values were less than 0.05, and the difference was statistically significant). These metabolites are mainly related to mitochondrial oxidative phosphorylation. We found that the production of ATP was significantly reduced after diacetylmorphine intervention, suggesting that diacetylmorphine may affect the oxidative phosphorylation process of mitochondria, interfere with ATP production and then affect the myocardial contractile function (Figures 7–9).

### 3.5 Action potential of myocardial cells

**Figure 10:**
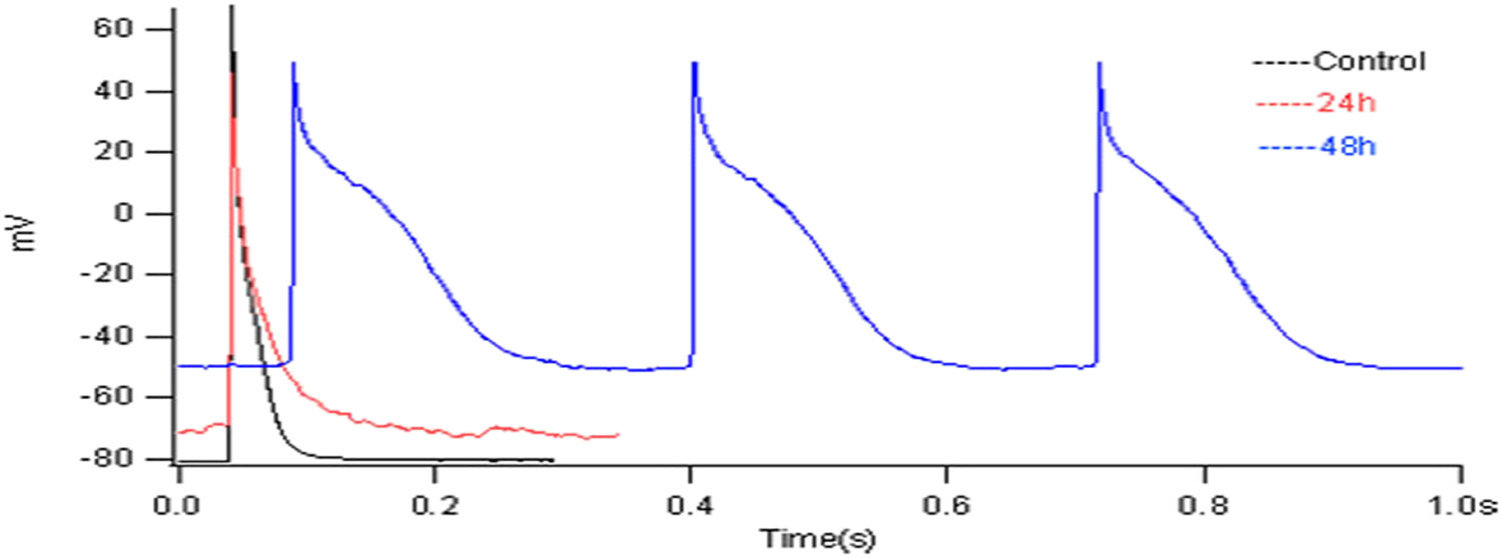
Action potential of cardiomyocytes in different groups. Action potential of cardiomyocytes in control group, heroin intervened for 24 and 48 h.

**Figure 11:**
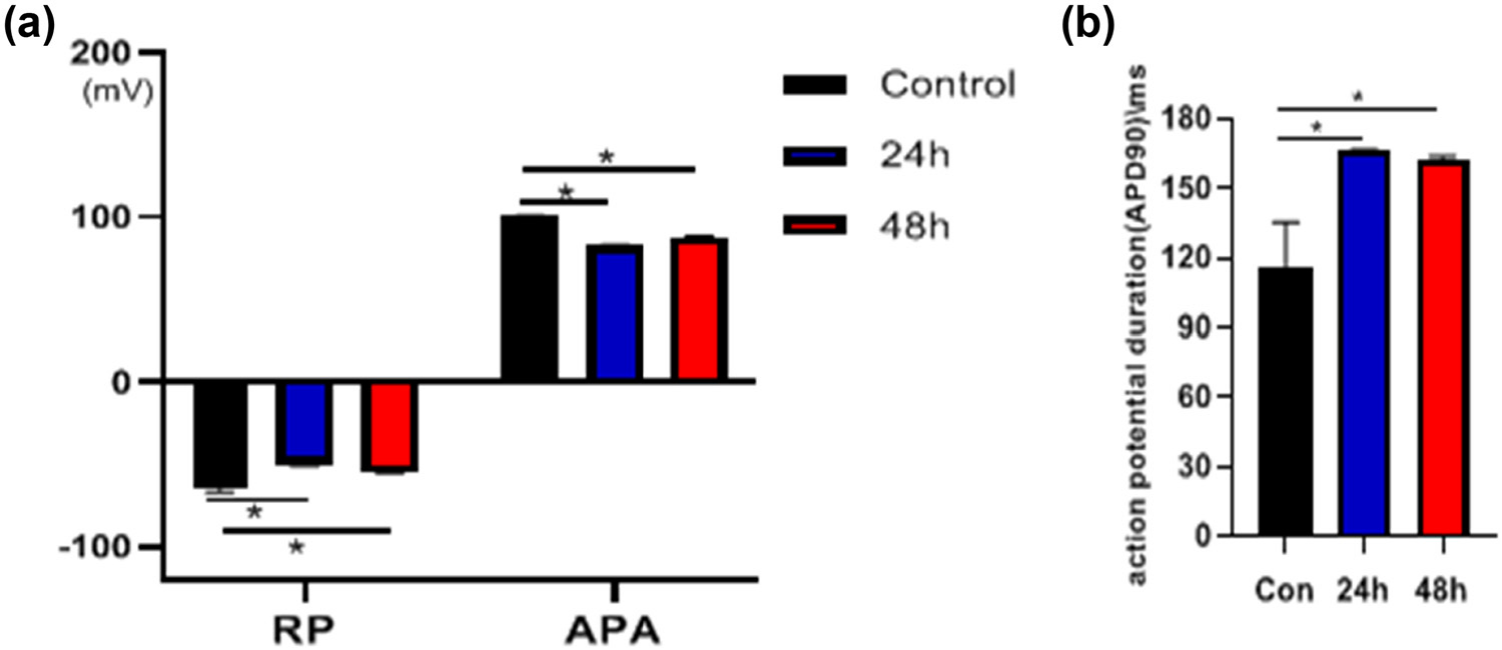
The changes in action potential in control group and 10^−4^ mol/L heroin intervention 24 and 48 h group. (a) RP and APA values of each group; (b) Statistical chart of APD for each group.

After 7 days of isolated culturing, the cardiomyocytes of the neonatal rats were treated with 10^−4^ mol/L heroin for 24 and 48 h, and the action potential was significantly changed compared with the N group. Compared with the normal control group, after the action of 10^−4^ mol/L heroin for 24 h, the APA and RP significantly decreased, and the APD was significantly prolonged, with the results being statistically significant (*p* < 0.05) (Figures 10 and 11). After 48 h of action, the negative value of the RP lowered, the APA significantly decreased, and the APD was significantly prolonged, with statistically significant results (*p* < 0.05) (Figures 10 and 11). The results showed that 10^−4^ mol/L heroin could change the AP of cardiomyocytes, decrease the RP and APA, increase the APD, and increase the APD with the extension of the action time.

### 3.6 L-type calcium channel current of cardiomyocytes

**Figure 12:**
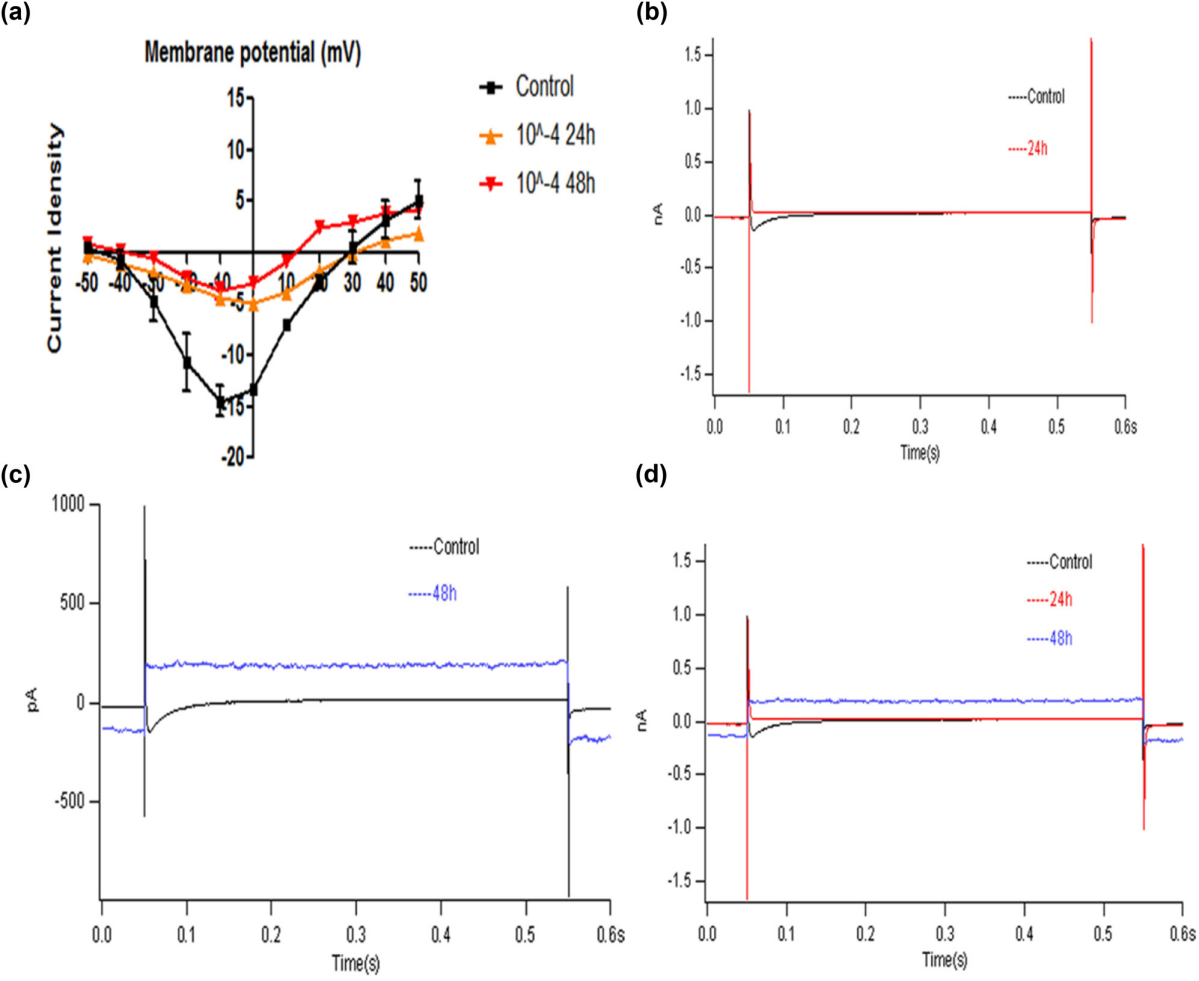
Changes in *I*_ca-L_ *I*−*V* curve and Ca^2+^ current intervened by heroin. (a) Changes in *I*_ca-L_ *I*−*V* curve in 10^−4^ mol/L heroin intervened group at different times. (b) Ca^2+^ current of cardiomyocytes after 10^−4^ mol/L heroin intervention 24 h. (c) Ca^2+^ current of cardiomyocytes after 10^−4^ mol/L heroin intervention 48 h. (d) Ca^2+^ current of cardiomyocytes after heroin intervention in different times.

The activation potential of the normal control group was −40 mV, the maximum peak potential was −10 mV, and the inversion potential was +30 mV. The interference of 10^−4^ mol/L heroin for 24 h significantly up-shifted the current–voltage curve. The peak pA/pF decreased from −14.469 ± 3.611 to −4.966 ± 0.771 (*p* < 0.05). The current–voltage curve was significantly up-shifted by 48 h of interference, and the peak pA/pF decreased from −14.469 ± 3.611 to −3.757 ± 2.060 (*p* < 0.05). The membrane potential was plotted based on peak pA/pF, and the *I*–*V* curve of *I*_ca_ was plotted. After the interference of heroin, the Ca^2+^ current was significantly changed in the *I*–*V* curve compared with the control group, and *I*–*V* curve was significantly up-shifted, but the curve shape was unchanged (Table 4, Figure 12a–d), indicating that heroin only affected *I*_ca-L_ current but did not change the electrophysiological properties of the Ca^2+^ channel.

**Table 4:** Changes in *I*_ca-L_ current density in control group and 10^−4^ mol/L heroin intervention 24 and 48 h group (pA/pF) ( \bar{x}] ± *s*, *n* = 6)

Voltage clamping	Con group	10^−4^ mol/L, 24 h	10^−4^ mol/L, 48 h
−50 mV	0.463 ± 0.919	−0.114 ± 0.369	0.801 ± 0.478
−40 mV	−0.678 ± 2.175	−1.034 ± 0.815	0.123 ± 0.643
−30 mV	−4.702 ± 4.855	−1.908 ± 0.400	−0.633 ± 0.777
−20 mV	−10.683 ± 6.695	−3.258 ± 0.750	−2.471 ± 0.785
−10 mV	−14.469 ± 3.611	−4.461 ± 0.270*	−3.757 ± 2.060*
0 mV	−13.322 ± 0.276	−4.966 ± 0.771*	−3.081 ± 1.502*
10 mV	−6.952 ± 1.227	−3.875 ± 0.974*	−0.875 ± 0.550*
20 mV	−2.897 ± 1.460	−1.852 ± 1.260	2.468 ± 1.190
30 mV	0.459 ± 3.810	−0.098 ± 0.929	3.027 ± 0.490
40 mV	3.188 ± 4.457	1.207 ± 0.549	3.789 ± 0.359
50 mV	5.114 ± 4.392	1.992 ± 0.187	4.233 ± 0.211

Vs: Compared with Con group, *p < 0.05.

## 4 Discussion

Heroin (chemical name: diacetylmorphine) is an opiate, a naturally occurring alkaloid extracted from the seedpod of the *Papaver somniferum*. After long-term use, it will cause not only serious damage to the immune function but also damage vital organs, such as the heart, brain, liver, and kidney [6]. The hypothesis of myocardial damage related to heroin includes striated muscle atrophy, hypoxia, acidosis, and vasoconstriction substance released by muscle necrosis or allergic reactions [7]. Therefore, further investigation regarding the exact mechanism of cardiovascular damage caused by heroin in the medical field is urgently needed. Diacetylmorphine can lead to the occurrence of arrhythmia and can cause the change in spontaneous beating frequency of cardiac myocytes, but the specific mechanism is still unclear and needs further investigation.

Ca^2+^ signaling plays a very important role in the opioid receptor-mediated signaling pathways in which many opioid receptor subtypes attaching to the myocardial cell membrane exist [8,9,10]. Opioid receptors are activated by opioids to protect against myocardial ischemia-reperfusion injury [11,12,13]. Ca^2+^, which acts as the second intracellular messenger, participates in various biological processes and activates multiple intracellular signaling molecules, which are more closely related to CaM, activated CaMKII, and calcineurin. CaMKII plays an important role in intracellular calcium homeostasis and regulation of the release and reabsorption of Ca^2+^ by sarcoplasmic reticulum receptor and sarcoplasmic reticulum Ca^2+^-ATPase and complete the excitation-contraction coupling of cardiomyocytes. When any process of calcium release, uptake, or storage is abnormal, will affect the Ca^2+^ circulation in myocardial cells, and then affect the Ca^2+^ balance in cardiomyocytes [8,9,14]. In the experiment of diacetylmorphine interfering with isolated myocardial cells, it was found that the decrease in spontaneous beating frequency of myocardial cells may be related to the depletion of Ca^2+^ stored in myocardial cells by diacetylmorphine, resulting in Ca^2+^ imbalance. However, the specific mechanism remains to be further explored.

Heroin, which belongs to the exogenous morphine opioid receptor agonist, competitively combines with the opioid receptor of the myocardial cell membrane to inhibit the formation and release of endogenous endorphin in the myocardium after entering the body, and it is different from synthetic morphine or opioid receptor agonists organ injury by its fat solubility, action local, action speed, withdrawal symptoms, response intensity, duration, and metabolic rate [10,15,16,17]. Therefore, it is unscientific to explain the toxic effects of heroin by using the study results on opioids. A previous study has found that an increase in spontaneous beat frequency in cardiomyocytes could be induced by heroin. The studies have shown that heroin could cause a significant increase in free Ca^2+^ concentration in cardiomyocytes depending on the dosage, and the isolated, cultured cardiomyocytes underwent calcium overload for a short period of time under the interference of heroin, but it could quickly drop to normal levels [18]. Verapamil, which significantly inhibits heroin-induced calcium overload in cardiomyocytes, can reduce cell death [19]. In animal experiments, the heart rate and amplitude of rats have been reduced by heroin, and this mechanism might be related to the storage of Ca^2+^ in depleted cardiomyocytes [20]. It indicated that heroin could transiently increase the levels of Ca^2+^ in cardiomyocytes, which could produce a transient positive inotropic effect. Additionally, a significant negative inotropic effect occurred after Ca^2+^ was depleted in the sarcoplasmic reticulum.

In this study, heroin could induce cardiomyocyte injury, and the patch-clamp technique detected cell electrophysiological function. It was found that the APD90 of cardiomyocytes was significantly extended by heroin. The APD increased, the negative value of RP and the APA decreased, while the L-Ca^2+^ channel current peak significantly decreased, with a statistically significant difference (*p* < 0.05).

*I*–*V* curve shifted up, but it did not change the shape of the curve. It indicated that the external calcium influx decreased, the repolarization time increased, and the APD significantly increased under the interference of heroin. The possible mechanism might be related to the depletion of Ca^2+^ stored in cardiomyocytes by heroin, and it is consistent with the results in heart rate and amplitude of electrocardiogram in rats induced by heroin in animal experiments [21]. In this study, the frequency of spontaneous beating in cardiomyocytes was slowed down by heroin. Heroin realized signal transduction by stimulating any opioid receptors on the surface of the myocardial cell membrane, whereas the mechanism of changes caused in the muscle force of myocardial cell remains to be further studied.

Calcium ions play an important role in the excitatory contraction coupling of the heart. The voltage-dependent L-type channel may be opened because of depolarization of the myocardial cell membrane and produces a transient calcium gradient to trigger the release of calcium ions from the sarcoplasmic reticulum [22]. The mechanisms by which diacetylmorphine induces myocardial toxicity are complex and multifactorial, including interference with cardiomyocyte energy metabolism and intracellular calcium processing, ROS production, neurohormonal stress, and induction of cardiomyocyte apoptosis. Under normoxic conditions, more than 95% of the ATP produced in the heart comes from oxidative phosphorylation in the mitochondria. Perturbations during ATP production may directly affect the contractile function of the myocardium [23]. In this study, the morphology and electrophysiological function of cardiomyocytes were changed under the interference of heroin. Cardiomyocyte transcriptomics and metabolomics also significantly varied, and myocardial contractile signaling pathways were also significantly altered, after diacetylmorphine intervention, many metabolites are significantly altered, these metabolites are mainly related to the mitochondrial oxidative phosphorylation. We found that the production of ATP was significantly reduced after diacetylmorphine intervention, suggesting that diacetylmorphine may affect the oxidative phosphorylation process of mitochondria, interfere with ATP production and then affect the myocardial contractile function, suggesting that heroin can significantly affect cardiomyocyte metabolic processes and then affect cardiac contractility. A series of activities, such as myocardial relaxation and contraction, were closely related to intracellular calcium ion contents. Heroin could induce abnormal changes in calcium ion contents and electrophysiological function in cardiomyocytes. Thus, calcium channels play a certain role in the process of myocardial rhythm abnormalities induced by heroin. In summary, heroin can damage the myocardium to a certain degree and cause changes in the action potential and L-type calcium channel, as well as the change in the electrophysiological function of cardiomyocytes. Our data are basically consistent with the results of animal experiments.

## 5 Conclusion

In this study, we found through transcriptomic and metabolomics studies that heroin can cause myocardial contraction and calcium channel abnormalities, damage the myocardium, and change the action potential and L-type calcium channel. These results will also provide molecular targets for finding and establishing a new therapeutic scheme for clinical diagnosis and prevention.
